# Validation of Targeted Relationships of Novel circRNA803/lncRNA MSTRG.19726–oar-let-7a–CPEB1 ceRNA Networks, Key to Follicle Development in Single-Litter and Multi-Litter Sheep Based on Whole-Transcriptome Sequencing

**DOI:** 10.3390/ijms26115161

**Published:** 2025-05-28

**Authors:** Bo Gu, Anqi Wang, Hang Liu, Xudong Liu, Huaizhi Jiang

**Affiliations:** 1 College of Life Science, Jilin Normal University, Siping 136000, China; 17743110597@163.com; 2 College of Animal Science and Technology, Jilin Agricultural University, Changchun 130118, China; 18043814927@163.com (A.W.); liuhang19960214@163.com (H.L.); xiang8118814477@163.com (X.L.)

**Keywords:** Ujumuqin sheep, Small-Tail Han sheep, ovary, ceRNA network

## Abstract

The objective of this study is to investigate the molecular regulatory mechanisms of non-coding RNA (ncRNA) during the developmental process of multi-litter sheep ovaries and identify key regulatory genes that enhance the reproductive capacity of sheep. This study selected Small-Tail Han sheep (multi-litter sheep) and Ujumuqin sheep (single-litter sheep) as comparative models, constructed the expression profiles of ncRNAs and mRNAs in ovarian tissues, identified differentially expressed (DE) lncRNAs, circRNAs, miRNAs, and mRNAs, and performed target gene prediction along with functional and signaling pathway enrichment analyses. Reproduction-related pathways were further screened to construct competing endogenous RNA (ceRNA) regulatory networks (lncRNA–miRNA–mRNA and circRNA–miRNA–mRNA). Finally, the dual-luciferase reporter gene assay system was employed to perform the functional validation of the relevant targeted regulatory effects. A comprehensive screening identified 411 DE lncRNAs, 322 DE circRNAs, 26 DE miRNAs, and 29 DEGs from the ovarian tissues of Ujumqin and Small-Tail Han sheep. The results of the Gene Ontology (GO) and Kyoto Encyclopedia of Genes and Genomes (KEGG) enrichment analyses demonstrated that the DE target genes were significantly enriched in pathways associated with cell dedifferentiation, the positive regulation of embryonic development, glycosaminoglycan biosynthesis, Hippo signaling, and other signaling pathways. To identify genes associated with reproductive processes, we performed differential expression screening followed by pathway enrichment analysis, which revealed significant enrichment in reproductive regulatory pathways. Based on these findings, we constructed a ceRNA regulatory network incorporating 22 DEGs, 17 DE lncRNAs, three DE circRNAs, and one DE miRNA. Our analysis revealed that oar-let-7a is involved in signaling pathways such as oocyte meiosis and Hippo, suggesting it may serve as a key miRNA regulating the trait of multiple offspring. The dual-luciferase reporter assay was employed to confirm that oar-let-7a directly targets and regulates the expression of *CPEB1*. Additionally, it was demonstrated that circRNA803 and lncRNA MSTRG.19726 function as molecular sponges to competitively bind and regulate oar-let-7a. These findings suggest that oar-let-7a mediates the expression of *CPEB1* via circRNA803 and lncRNA MSTRG.19726 sponge adsorption, thereby regulating the process of follicular dominance in sheep. The qRT-PCR method was employed to validate the expression patterns of nine randomly selected DEGs, and the results corroborated the reliability of the RNA-seq sequencing data. This study investigated the coordinated regulatory mechanism of DE ncRNAs and their corresponding target genes, identifying a ceRNA network, circRNA803/lncRNA MSTRG.19726-oar-let-7a-*CPEB1*, which plays a critical role in regulating the process of follicular dominance in sheep. These findings provide fundamental data for uncovering the reproductive potential of sheep and facilitate a comprehensive understanding of their reproductive characteristics, which hold significant guiding implications for enhancing reproductive efficiency.

## 1. Introduction

The apparent consumption of mutton among Chinese residents has increased annually since 2015, and the level of apparent consumption has surpassed the total domestic production of mutton annually since 2020 to meet domestic demand, thereby establishing itself as a net importer of mutton. Therefore, expanding the breeding scale of sheep and enhancing individual meat production capacity represent effective strategies to address the issue of self-sufficiency in mutton. Improving the reproductive efficiency of meat sheep is the optimal approach to achieving standardized meat sheep breeding. The reproductive efficiency of sheep is determined by the ovarian ovulatory output, which is closely associated with “follicular waves” during the estrous cycle—particularly the process of follicular dominance. Like other mammals, the ovine ovary undergoes a “wave” development process characterized by “recruitment—selection—dominance—maturation—ovulation” during the estrus cycle after reaching sexual maturity. Previous studies both domestically and internationally have demonstrated that ncRNAs, including lncRNA, circRNA, and miRNA, play crucial regulatory roles in the development of the mammalian ovary. Notably, lncRNAs and circRNAs function as “sponges” that adsorb miRNAs, reducing their activity and indirectly influencing the regulatory network of target gene mRNAs. This mechanism has made lncRNAs and circRNAs focal points in the transcriptional regulation mediated by ncRNAs [1,2,3]. The ovaries of pigs differentially express 775 lncRNAs during estrus and interestrus cycles, and by constructing ceRNA networks, 40 hub lncRNAs were screened to regulate 20 target genes associated with ovarian function and reproductive pathways through sponge adsorption of 32 miRNAs; this mechanism can also affect the maturation of ovarian oocytes and the synthesis and regulation of ovarian hormones in estrus [4]. It was found that lnc-MSTRG.7889/bta-miR-146a/*Smad4* was shown to contain ceRNA and regulated the apoptosis of ovarian granulosa cells in yaks [5]. Screening for differentially expressed lncRNAs, miRNAs, and mRNAs in the corpus luteum of various sheep breeds and constructing a ceRNA interaction network identified 44 mRNAs, seven lncRNAs, and seven miRNAs as potentially influencing luteal formation through the regulation of biological processes such as cell adhesion, cell differentiation, and lipid metabolism [6]. Studies have demonstrated that performing whole-transcriptome sequencing on granulosa cells and cumulus cells within yak follicles, as well as constructing an lncRNA–miRNA–mRNA interaction network, can offer a robust theoretical foundation for investigating the proliferation and differentiation mechanisms of granulosa cells and the developmental processes of oocytes [7]. MSTRG.28645 was also found to be a key gene in the ceRNA network and is involved in the steroid biosynthesis signaling pathway, regulating sheep fertility [8]. Mapping the expression profiles of differential lncRNAs and mRNAs in the ovarian tissues of Hu sheep and Mongolian sheep identified the key gene *MMP2,* which may regulate the reproductive performance of sheep through the reproductive axis [9]. Analysis of the ceRNA network in ovarian tissues during the follicular and luteal phases in both singlet and multi-litter sheep identified circLTBP1 as a potential ceRNA regulating gene transcription and influencing follicular development [10]. Therefore, the analyzing the regulatory mechanism underlying the ceRNA network during ovarian development has important theoretical implications for improving the reproductive efficiency of animals.

Small-Tail Han sheep is one of the few multi-litter sheep breeds in the world. It is famous for its high fertility and plays a unique role in the development of China’s meat sheep industry. Ujumuqin sheep, a branch of Mongolian sheep, is an ancient and primitive sheep breed in China whose reproductive rate is limited due to low reproductive ability and seasonal estrus [11]. In this study, Small-Tail Han sheep (multi-litter sheep) and Ujumuqin Sheep (single-litter sheep), which are widely raised in Jilin Province, were analyzed to obtain RNA-seq data during estrus, obtain comprehensive mRNA, miRNA, lncRNA, and circRNA expression profiles of sheep ovaries, and construct a ceRNA network. Then, the key genes and molecular regulatory mechanisms controlling ovine ovarian development were analyzed. The findings of this study offer a solid theoretical foundation for comprehensively understanding the reproductive biological traits of sheep and effectively guiding the application of advanced breeding techniques, such as the development of new breeds of multi-litter sheep and promoting frequent lambing in sheep.

## 2. Results

### 2.1. Quality Control and Comparative Analysis to Reference Genome

After sequencing, the raw reads of ovarian tissue samples from Ujumuqin sheep (Uo1, Uo2, and Uo3) and Small-Tail Han sheep (So1, So2, and So3) were filtered. The data are shown in Table 1 and Table 2. The proportion of Q20 in each lncRNA and circRNA sample was above 99%, the proportion of Q30 was above 97%, and the GC content was between 44.5% and 46%. Moreover, the rate of alignment rate to the reference genome exceeded 92%. The Q20 and Q30 scores for all small RNA samples were consistently above 97%, with the GC content ranging from 51.05% to 51.78%. The sequencing data demonstrated high quality, passed all quality control metrics, and were thus suitable for subsequent analyses.

### 2.2. Screening and Identification of lncRNAs, circRNAs, and miRNAs

**Figure 1:**
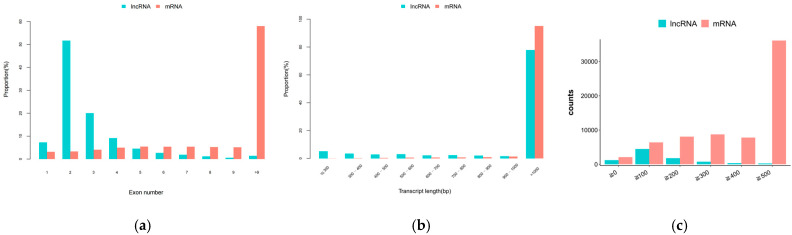
Identification of lncRNAs and mRNAs in ovarian tissues of Small-Tail Han sheep and Ujumqin sheep: (**a**) lncRNA and mRNA exon numbers; (**b**) lncRNA and mRNA transcript lengths; (**c**) lncRNA and mRNA ORF length.

A total of 8250 lncRNAs were identified in the ovarian tissues of Ujumuqin and Small-Tail Han sheep, among which 4395 were known lncRNAs and 3855 were novel lncRNAs. Further statistical analysis was performed to determine the exon number, transcript length, and ORF characteristics of the lncRNA and mRNA. The results showed that most of the lncRNAs had two exons, significantly fewer than the mRNAs (Figure 1a). The overall trends of the lncRNA and mRNA sequence lengths were similar, mainly >1000 bp (Figure 1b). The longest ORFs in the lncRNAs were 100–200 bp, significantly shorter than those of the mRNAs (Figure 1c).

**Figure 2:**
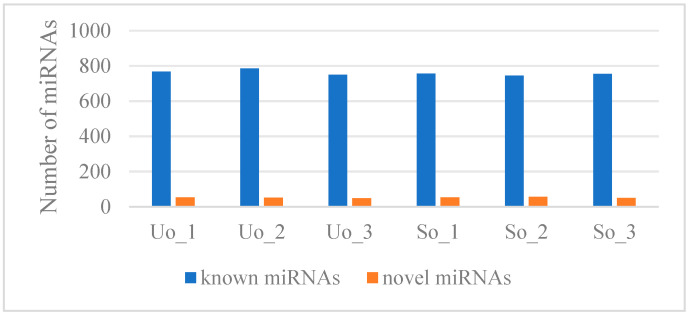
Number of known and novel miRNAs.

Comparative analysis of the miRNAs identified 760 known and 53 novel miRNAs (Figure 2).

**Figure 3:**
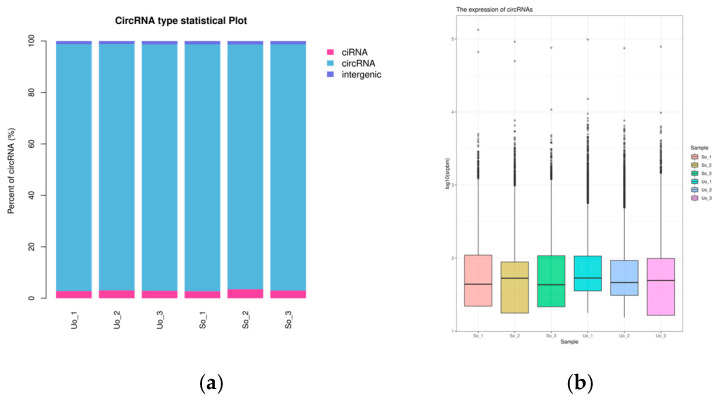
The circRNA types and expression statistics: (**a**) circRNA type statistics; (**b**) circRNA expression distribution map.

Analysis of circRNAs identified results showed that 14,015, 12,880, 11,645, 9425, 12,323, and 10,507 circRNAs in Uo1, Uo2, Uo3, So1, So2, and So3, respectively. Most of the circRNAs were of whole-exon type, and their expression levels were similar, as shown in Figure 3.

### 2.3. Differential Expression Analysis and Target Gene Prediction

**Figure 4:**
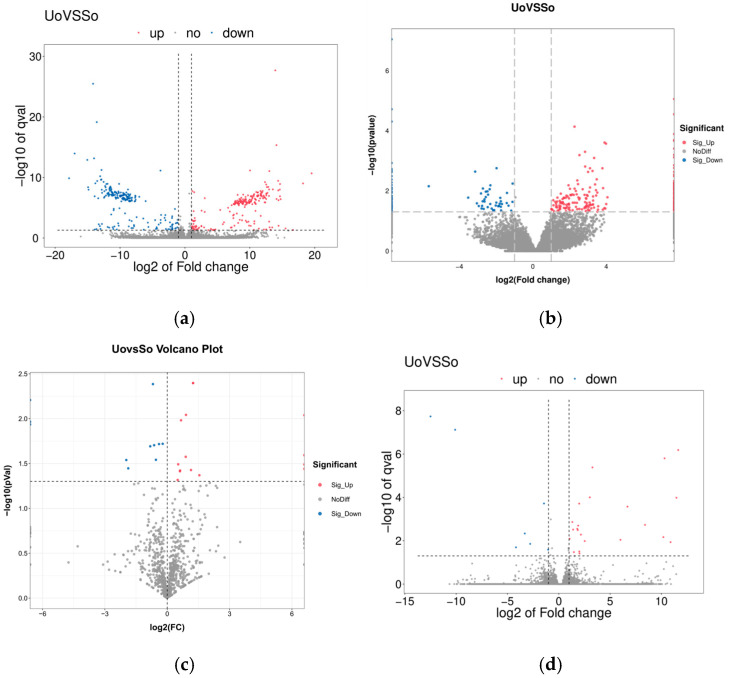
The Volcano plots of lncRNAs, circRNAs, miRNAs and mRNAs that were differentially expressed (DE) between Uo and So: (**a**) DE lncRNAs; (**b**) DE circRNAs; (**c**) DE miRNAs; (**d**) DE mRNAs.

A total of 411 DE lncRNAs were identified in the ovarian tissues of Ujumuqin sheep vs. Small-Tail Han sheep; of these, 212 were upregulated and 199 were downregulated (Figure 4a). Additionally, 322 DE circRNAs were identified, of which 226 were upregulated and 96 were downregulated (Figure 4b). A total of 26 DE miRNAs were also identified, of which 15 were upregulated and 11 were downregulated (Figure 4c). Finally, 29 DEGs were identified, of which 22 were upregulated and seven were downregulated (Figure 4d).

### 2.4. GO and KEGG Enrichment Analysis

**Figure 5:**
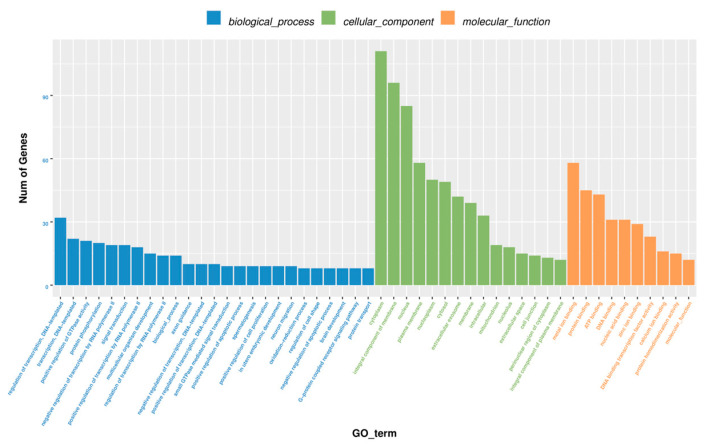
GO function enrichment analysis.

GO functional enrichment analysis was performed to explore the key genes involved in ovarian development. The target genes were significantly enriched in 179 GO terms, including biological processes such as the inositol phosphate metabolic process, cell dedifferentiation, and positive regulation of embryonic development; cellular components such as mitochondrial membrane constituents and cytosolic ribosomes; and molecular functions like calcium-transporting ATPase activity and kinase activator activity. The terms were sorted, in descending order, by the number of annotated differentially expressed genes; then, the top 25, top 15, and top 10 terms were selected for graphical presentation (in Figure 5).

**Figure 6:**
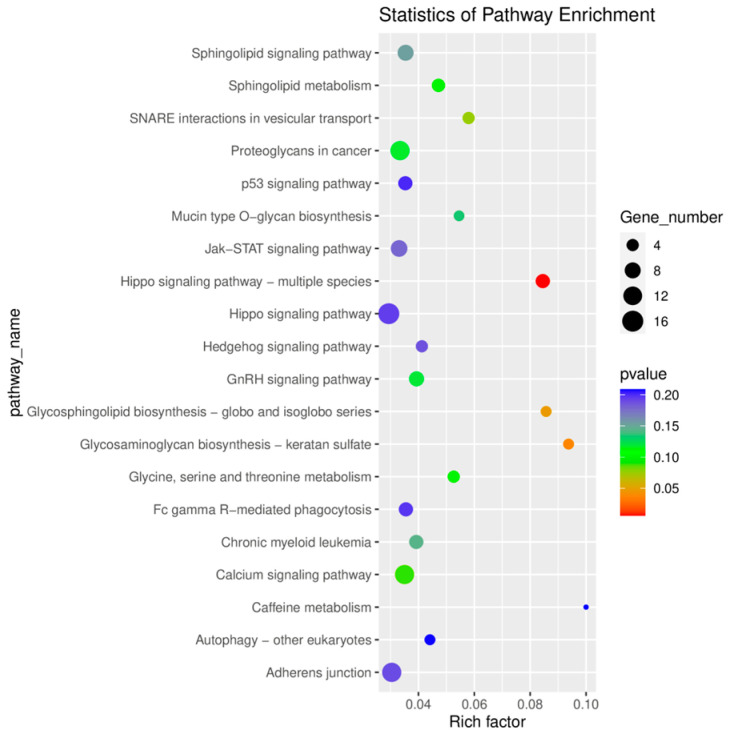
KEGG pathway enrichment analysis.

KEGG enrichment analysis revealed significant enrichment of signaling pathways, including the Hippo signaling pathway, glycosaminoglycan biosynthesis, and glycosphingolipid biosynthesis. The top 20 signaling pathways are displayed in Figure 6.

### 2.5. ceRNA Network Analysis

**Figure 7:**
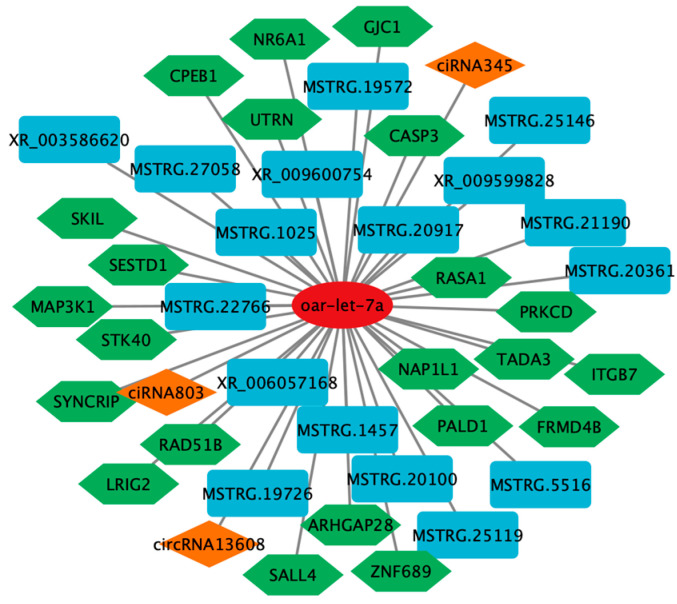
Diagram of the ceRNA regulatory network.

With DE miRNAs as the core, ceRNA networks were constructed by combining DE lncRNAs and DE circRNAs, which have a sponge adsorption effect on miRNAs, and DE mRNAs targeted by DE miRNAs. The ceRNA networks consisted of lncRNA–miRNA–mRNA and circRNA–miRNA–mRNA. ceRNA networks (including 17 DE lncRNAs, three DE circRNAs, one DE miRNA, and 22 DE mRNAs) involved in reproductive pathways were visualized (Figure 7) by screening for key miRNAs that play a common role in the network to form lncRNA/circRNA/–miRNA–mRNA and conducting bioinformatics analysis results. (Other ceRNA networks are shown in Supplementary Table S2.)

### 2.6. qRT-PCR Validation of Sequencing Data

**Figure 8:**
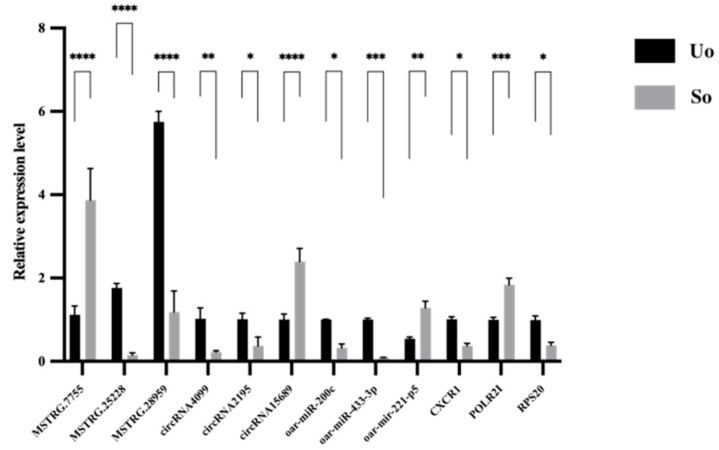
qRT-PCR validation of the RNA-seq data for lncRNAs, circRNAs, miRNAs, and mRNAs. (* *p* < 0.05, ** *p* < 0.01, *** *p* < 0.001, **** *p* < 0.0001).

The relative quantification results are shown in Figure 8. The expression level of MSTRG.7755, circRNA15689, oar-miR-211-p5, and *POLR*21 in the ovaries of Small-Tail Han sheep were significantly higher than in Ujumuqin sheep (*p* < 0.01). However, the expression levels of MSTRG.25228, MSTRG.28959, circRNA4099, and oar-miR-433-3p in the ovaries of Small-Tail Han sheep were significantly lower than in Ujumuqin sheep (*p* < 0.01). Furthermore, the expression levels of circRNA2195, oar-miR-200c, *CXCR*1, and *RPS*20 in the ovaries of Small-Tail Han sheep were significantly lower than in Ujumuqin sheep (*p* < 0.05). The qRT-PCR results of these lncRNAs, circRNAs, miRNAs, and mRNAs were similar to the RNA-seq results, indicating that the sequencing results were accurate and replicable.

### 2.7. Double Luciferase Reporter Gene Assay

**Figure 9:**
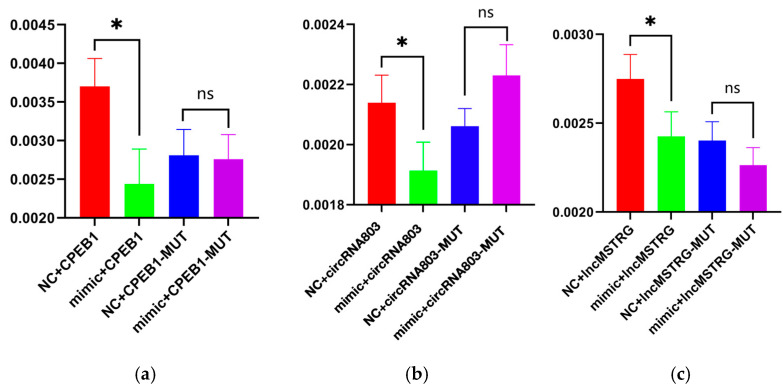
Dual-luciferase assay verification of the direct target relationship between oar-let-7a and CPEB1 (**a**), oar-let-7a and circRNA803 (**b**), and oar-let-7a and lncMSTRG.19726 (**c**). (* *p* < 0.05, ns not significant).

The results of verifying the binding of oar-let-7a to *CPEB1* showed that the oar-let-7a mimic significantly reduced luciferase expression of wild-type *CPEB1*, and there was no effect after mutating the binding sequences (Figure 9a). These results indicate that oar-let-7a can bind to the *CPEB1* sequence and has a targeted regulatory relationship. The results verifying the binding of oar-let-7a to circRNA803 also showed that the oar-let-7a mimic significantly reduced luciferase expression of wild-type circRNA803, with no effect after mutation of binding sequences (Figure 9b). These results indicate that oar-let-7a can bind to the circRNA803 sequence. Comprehensive analysis showed that oar-let-7a can mediate the expression of *CPEB1* through a circRNA803 sponge, forming the ceRNA regulatory network circRNA803–oar-let-7a–*CPEB1*. On the other hand, the results verifying the binding of oar-let-7a to lncRNA MSTRG.19726 showed that the oar-let-7a mimic significantly reduced luciferase expression of wild-type MSTRG.19726, and mutating the binding sequence had no effect (Figure 9c). These results indicate that MSTRG.19726 can sponge-adsorb oar-let-7a to affect the expression of *CPEB1* and form the ceRNA network of MSTRG.19726–oar-let-7a–*CPEB1*. In summary, circRNA803 and the lncRNA MSTRG.19726 are both involved in the sponge adsorption of oar-let-7a, forming the circRNA803/MSTRG.19726–oar-let-7a–*CPEB1* regulatory network.

## 3. Discussion

Improving the lambing rate and shortening the breeding interval of mutton sheep has always been the unremitting pursuit of the mutton sheep industry. Although a lot of work has been conducted to improve the breeding characteristics and reproductive efficiency of mutton sheep in China, the low reproductive efficiency has not been fundamentally solved. Analysis of the molecular regulation mechanism of ovarian development will be the key to promoting the efficient application of reproductive biology and modern breeding techniques in meat sheep production. Increasing research has found that ncRNAs play a unique role as participants in gene network regulation during ovarian development, which has important theoretical significance for elucidating the reproductive mechanism in sheep. LncRNAs, as long-chain endogenous ncRNAs, can regulate the expression of related genes through cis-or trans-action and act as “miRNA sponges” to negatively regulate the expression of target genes [12]. Studies have found that lncRNAs are involved in a variety of reproductive processes in animals, including ovarian development [13], pregnancy maintenance [14], oocyte maturation [15], and sex hormone secretion [16]. circRNAs are a special class of RNA that can act as ceRNAs and constitute the circRNA–miRNA–mRNA network, mediate the information pathway associated with ovarian development and the secretion of reproductive hormones, and participate in the regulation of early embryonic development to play their regulatory role [17,18,19,20]. Therefore, studying the expression and function of ceRNA in the ovaries of different breeds of sheep is of great biological significance.

In this study, ncRNA and mRNA expression profiles were generated for the ovaries of Ujumuqin and Small-Tail Han sheep during the estrus period. Bioinformatics analysis revealed that the significantly enriched Hippo signaling pathway may play a crucial role in regulating ovarian development. All components of the Hippo signaling pathway are expressed in the ovaries and play a crucial role in regulating the proliferation of mammalian germ cells, follicular development, and luteal formation [21]. Further studies demonstrated that *CPEB1* is significantly enriched in this signaling pathway. By integrating the constructed ceRNA network and the dual-luciferase reporter gene assay system, it was conclusively determined that oar-let-7a directly regulates the expression of *CPEB1* through targeted interaction. Additionally, circRNA803 and the lncRNA MSTRG.19726 have a sponge-like adsorption effect on oar-let-7a. It is evident from this analysis that the circRNA803/lncRNA MSTRG.19726–oar-let-7a–*CPEB1* ceRNA network plays a pivotal role in the development of sheep ovaries.

The *CPEB1* gene is an important member of the cytoplasmic polyadenylation element binding protein (CPEB) family. It recruits translation inhibitors to the CPE in the 3′ untranslated region of its target mRNA and binds to its specific sequence (UUUUUAU) to regulate the polyadenylation of mRNA, thereby regulating the translation of mRNA. Current results from multiple research studies show that *CPEB1* is highly expressed in oocytes in the ovary, suggesting that it plays an important role in oogenesis and the normal development of the ovary. *CPEB1* is essential in the meiosis process in female germ cells following the cratinous phase [22]. In addition, numerous oocyte and ovarian abnormalities were observed in both adolescent (6–8 weeks) and older transgenic (TG) mice generated by specifically depleting *CPEB1* mRNA after the coarse-line phase [23]. These results suggest that *CPEB1* plays an essential role in the early stages of oogenesis and oocyte maturation in mice. In human studies, heterozygous deletion of the *CPEB1* gene results in haploinsufficiency, which is associated with the development of primary ovarian insufficiency (POI) [24]. Furthermore, research has demonstrated that human aging is closely associated with functional impairments of *CPEB1* [25]. The level of *CPEB1* protein in aged oocytes decreases by approximately 50%, potentially leading to translational defects in maternal mRNA within aging oocytes. This may result in the observed reduction in oocyte quantity during maternal aging and compromised developmental competence. These changes contribute to a decline in oocyte quality and overall fertility. Research findings have demonstrated the interaction between *CPEB1* and c-mos mRNA in zebrafish oocytes [26]. Other studies have reported that zebrafish cyclin B1 mRNA undergoes *CPEB1*-mediated cytoplasmic polyadenylation via the CPE sequence and is subsequently activated during oocyte maturation [27]. All these findings collectively demonstrate that *CPEB1* plays an indispensable role in the process of oocyte maturation. Studies of livestock animals have demonstrated that *CPEB1* plays a critical role in the synthesis of cyclin B within porcine oocytes and the resumption of meiosis [28]. *CPEB1* plays a crucial role in promoting the development of bovine follicles [29]. Furthermore, the overexpression of *CPEB1* significantly enhances the proliferation of goat granulosa cells, whereas its effectively suppresses this process; these findings suggest that *CPEB1* serves as a critical regulator of the proliferation of goat granulosa cells [30]. The aforementioned research findings collectively demonstrate that *CPEB1* plays a pivotal role in the biological functions of oocytes and granulosa cells. In conclusion, *CPEB1* may influence follicular development and consequently regulate the multiple-litter trait in sheep by participating in these biological processes.

Let-7a is an essential member of the let-7 family and exhibits specific expression in granular cells. A substantial body of evidence from the ongoing research has confirmed that let-7a exerts significant regulatory effects over various biological processes, including animal growth and development, cell differentiation, and apoptosis. A study of pigs found that the expression level of let-7a in follicular granulosa cells is inversely proportional to the degree of follicular atresia, indicating that let-7a plays a key role in granulosa cell apoptosis and subsequently participates in follicular development and atresia regulation in pigs [31]. Investigation of Low-Density small Extracellular Vesicles (LD-sEVs) within the follicular fluid of bovine small follicles revealed that let-7i suppresses granulosa cell apoptosis by targeting *FasL*, indicating that it plays a crucial role in regulating the normal development of follicles [32]. Analysis of the reproductive performance of sheep revealed that the number of reads undergoing base editing at the oar-let-7a site was relatively large, affecting the number of lambs born [33]. The key gene miRNA-let-7b was identified when kisspeptin treatment was applied to the ovarian granulosa cells of Tan sheep. Subsequent investigations demonstrated that let-7b suppresses steroid hormone secretion and the proliferation of ovarian granulosa cells in Tan sheep by binding to *ITGB7* while also promoting their apoptosis [34].

In summary, the findings of this study are in agreement with the aforementioned research results, suggesting that oar-let-7a plays a role in regulating sheep ovarian development by targeting and modulating *CPEB1*. However, the precise mechanism of action remains to be elucidated. In conjunction with prior research outcomes, this study hypothesizes that oar-let-7a may influence ovarian development by regulating the proliferation and apoptosis of granulosa cells, thereby serving as a potential candidate for further validation studies.

## 4. Materials and Methods

### 4.1. Sample Collection

Study animals were obtained from the Guofeng livestock breeding sheep farm (Songyuan City, Jilin Province, China). Three menopausal ewes each of interestrus Small-Tail Han sheep and Ujumuqin sheep with the same feeding conditions and good development were selected for concurrent estrus treatment. The ewes were implanted with a progesterone vaginal suppository for 14 days and then injected with pregnant horse serum gonadotropin. The following day, estrus detection was performed using a teaser ram, and mounting behavior was considered indicative of estrus. Ovarian tissue blocks (3–5 cm^2^) were collected after slaughter and immediately placed in liquid nitrogen for the extraction of total RNA.

### 4.2. Total RNA Extraction and Library Construction

The TRIzol method was used to extract total RNA from ovarian tissue. Concentration and integrity tests were then conducted and an RNA library constructed after a quality inspection. The ribosomal RNA depletion (rRNA) method was used to construct strand-specific libraries (fr-firstrand) for lncRNAs and circRNAs. Samples that passed quality inspection were the sequenced using Illumina Novaseq^TM^ 6000 (Illumina Inc., San Diego, CA, USA). The sequence read length was 2 × 150 bp (PE150). An sRNA sequencing library was constructed using TruSeq Small RNA Sample Prep Kits (Illumina, San Diego, CA, USA) for miRNAs. After quality inspection, Illumina HiSeq 2000/2500 (Illumina lnc., San Diego, CA, USA) was used for sequencing. The sequence read length was 50 bp (single-ended).

### 4.3. Quality Control and Reference Genome Alignment

Cutadapt [35] was used to preprocess the lncRNA and circRNA sequence data, and those that failed quality control analysis were filtered to obtain clean data. Hisat2 [36,37,38] was used for lncRNA comparison with the reference genome, and the genes were analyzed separately based on gene location information specified using genome annotation. The transcription assembly software StringTie (2.1.6) was used to assemble and screen reads under the following conditions: transcript length greater than or equal to 20 bp, coverage of 3 or more transcriptional reads, and 1 or more exons per transcript. The coding ability of the remaining transcripts was predicted using CPC and CNCI. The structural characteristics were compared with those of mRNAs. After reference genome comparison with circRNAs, reverse shear points (BSJ) were identified in reads with no reference genome comparison, and the intersection of CIRCexplorer2 [39] and CIRI [40] was used to identify circRNAs.

miRNA data were analyzed using ACGT101-miR (v4.2), and 3′ splicers and junk sequences were removed. The screening length ranged from 18 to 26 nt. Reads were obtained after comparing and filtering the data using various RNA databases, including mRNA, RFam, and Repbase. miRNA identification was performed by comparing the precursor to the genome.

### 4.4. Differential Expression Analysis and Target Gene Prediction

DEseq2 was used to compare and analyze the differences in the expression levels of lncRNA transcripts; significance levels were set at |log_2_fc| ≥ 1 and *p* < 0.05. Furthermore, target genes were predicted for the screened DE lncRNAs. LncRNAs function mainly by regulating the expression of their neighboring genes; therefore, the upstream and downstream 100 kb of mRNAs and lncRNAs can be cis-regulated and predicted based on their location, with the obtained genes being the homeopathic regulatory target genes of lncRNAs. edgeR was used to analyze the differences in circRNA expression, with significance set at |log_2_fc| ≥ 1 and *p* < 0.05. The threshold for significantly differentially expressed miRNA was *p* < 0.05. Further, TargetScan (v5.0) and miRanda (v3.3a) were used for target gene prediction for significantly differentially expressed miRNAs.

### 4.5. ceRNA Network Construction and Bioinformatics Analysis

The target genes predicted by key miRNAs were annotated, and their signaling pathways were analyzed using the Gene Ontology database (http://geneontology.org/) (accessed on 20 November 2024) and the KEGG database (http://www.genome.jp/kegg/) (accessed on 20 November 2024). The reproduction-related pathways were further screened, and the key miRNAs were determined.

An important function of lncRNAs and circRNAs is adsorbing miRNAs, similar to a sponge. Rargeting and binding miRNAs results in changes in the expression of the target genes regulated by miRNAs; these changes are ultimately reflected in the expression levels of proteins. miRNAs also play a core role in the ceRNA regulatory network. LncRNA–miRNA–mRNA and circRNA–miRNA–mRNA relationship pairs were obtained, the miRNAs playing the core regulatory role were selected, and the regulatory network diagram was constructed using Cytoscape software (3.10.1) to visualize the regulatory relationships.

### 4.6. RNA-Seq Data Validation Using qRT-PCR

Three lncRNAs, circRNAs, miRNAs, and mRNAs were randomly selected for qRT-PCR verification using the SYBR Green incorporation method. (The primer sequences are shown in Supplementary Table S1). The relative quantitative results were determined using the 2^−ΔΔCt^ method, and SPSS ver.18.0 was used for a one-way statistical analysis of variance and significance testing.

### 4.7. Cell Culture

HEK-293T cells were resuscitated and cultured in DMEM containing 10% fetal bovine serum, 1.5 mM L-glutamine, 100 U/mL penicillin, and 100 μg/mL streptomycin in a 5% CO_2_-saturated humidity incubator at 37 °C. Adherent cells were passaged daily with 0.05% trypsin–EDTA.

### 4.8. Cell Transfection

A clean, sterile centrifuge tube was taken, and DMEM culture medium containing no antibiotics and serum was added. Then, 1 μg/μL of plasmid and 40 pmol mimics were added for co-transfection and gently blown and mixed with a gun. Subsequently, 1.6 μL of transfection reagent was added and the mixture incubated at room temperature for 20 min, after which 50 μL was added to each well. Cells were collected after transfection for 24 h.

The experimental design was divided into the following groups:

NC: *CPEB1* + NC-mimic, circRNA803 + NC-mimic, and lncRNA MSTRG.19726 + NC-mimic;
Mimic: *CPEB1* + mimic, circRNA803 + mimic, and lncRNA MSTRG.19726 + mimic;
NC + MUT: *CPEB1*-MUT + NC-mimic, circRNA803-MUT + NC-mimic, and
lncRNA MSTRG.19726-MUT + NC-mimic;
Mimic + MUT: *CPEB1*-MUT + mimic, circRNA803-MUT + mimic, and lncRNA MSTRG.19726-MUT + mimic.

### 4.9. Double Luciferase Reporter Detection

After lysis, the cells were transferred to an opaque 96-well plate, and then 100 μL of Luciferase Reaction Reagent, balanced at room temperature, was added to the plate. The activity of the firefly luciferase reporter gene was detected by horizontal mixing. Subsequently, 100 μL of Luciferase Reaction Reagent Il, balanced at room temperature, was added to the plate and mixed with horizontal shaking to detect the activity of the luciferase reporter gene in a sea kidney. Data acquisition was followed by statistical analysis of the recorded readings.

## 5. Conclusions

In this study, we constructed competing endogenous RNA (ceRNA) networks during the estrus period in the ovaries of multi-litter (Small-Tail Han sheep) and single-litter (Ujumuqin sheep) sheep. We identified the miRNA oar-let-7a as a key regulatory factor; additionally, we identified its target gene *CPEB1*. We further confirmed that oar-let-7a regulates the expression of *CPEB1* via adsorption by circRNA803 and MSTRG.19726 sponges using the dual-luciferase reporter system. These findings indicate that the circRNA803/lncRNA MSTRG.19726–oar-let-7a–*CPEB1* regulatory network plays a core role in follicular dominance in sheep, leading to multiple pregnancies. This discovery provides fundamental data for elucidating the molecular regulatory mechanism underlying ovarian development in multi-litter sheep and offers a theoretical foundation for further investigation into the regulatory mechanisms underlying high reproductive capacity in sheep.

## Figures and Tables

**Table 1:** Quality of lncRNA and circRNA sequencing data output.

Sample	Raw Data	Valid Data	Mapped Reads	Q20%	Q30%	GC Content%
Uo_1	92,231,148	89,013,622	83,650,569 (93.98%)	99.81	98.31	44.50
Uo_2	95,944,996	92,841,144	86,837,283 (93.53%)	99.81	98.24	46.00
Uo_3	89,602,454	86,517,238	81,071,259 (93.71%)	99.79	98.16	46.00
So_1	74,106,726	71,910,234	66,210,782 (92.07%)	99.85	98.05	45.00
So_2	90,402,358	87,502,414	81,890,808 (93.59%)	99.82	97.99	44.50
So_3	88,799,682	86,496,168	81,411,487 (94.12%)	99.86	98.02	45.00

**Table 2:** Quality of miRNA sequencing data output.

Sample	Raw Data	Valid Data	Mapped Reads	Q20%	Q30%	GC Content%
Uo_1	10,900,124	6,614,309	6,564,040 (99.24%)	99.26	97.16	51.08
Uo_2	11,914,260	6,735,685	6,679,105 (99.16%)	99.36	97.49	51.20
Uo_3	11,923,394	7,321,518	7,265,874 (99.24%)	99.33	97.42	51.10
So_1	11,979,084	7,224,597	7,153,073 (99.01%)	99.26	97.11	51.19
So_2	11,528,577	6,679,309	6,629,214 (99.25%)	99.29	97.16	51.05
So_3	12,785,226	5,824,807	5,763,647 (98.95%)	99.16	97.11	51.78

## Data Availability

The raw RNA-seq data generated in this study have been deposited in the NCBI Sequence Read Archive (SRA) under BioProject accession number PRJNA1253307.

## Supplementary Materials

The supporting information can be downloaded at https://www.mdpi.com/article/10.3390/ijms26115161/s1.

## Abbreviations

The following abbreviations are used in this manuscript:

DE	differentially expressed
ncRNA	non-coding RNA
ceRNA	competing endogenous RNA
GO	Gene Ontology
KEGG	Kyoto Encyclopedia of Genes and Genomes
