# Supplementary material for: Validation of Targeted Relationships of Novel circRNA803/lncRNA MSTRG.19726–oar-let-7a–CPEB1 ceRNA Networks, Key to Follicle Development in Single-Litter and Multi-Litter Sheep Based on Whole-Transcriptome Sequencing
Source: Int J Mol Sci. 2025 May 28;26(11):5161. doi: 10.3390/ijms26115161 (PMC12154228; doi:10.3390/ijms26115161)
Supplement: Supplementary file 1 [file ijms-26-05161-s001.zip › ijms-3646259-supplementary.pdf]

Table S1 qRT-PCR primers sequence information

Gene name	Sequence information	Length/bp	Annealing temperature/℃
β-actin	F: GATCTGGCACCACACCTTCTA	115	60
	R: GATCTGGGTCATCTTCTCACG		
MSTRG.7755	F: GCGTTGCTCTGTTCTTTATCTCCTG	144	60
	R: ACCTGTGCTGTCTGAGTCTTGTG		
MSTRG.25228	F: CCTGATCTTGTCCATCGTCCTATCC	123	60
	R: GGGAACTGACCAAGGATGATAAGGG		
MSTRG.28959	F: TGCTAAGCCTATGCCACTAGATGC	128	60
	R: CTCAAAGTCACCAGTTTCCCAAAGG		
circRNA4099	F: TGGAGCCTGTAATAACATGTGAC	259	60
	R: AGTTCCGCTGTGTTTGCATC		
circRNA2195	F: GGCTGGAGGTTTAAGAAGCG	125	60
	R: TTGAGGAAGGCAGACAGGTC		
circRNA15689	F: GTGAGCAAATTGTGGAAGTTGGA	102	60
	R: GGCTTCAAATCTCATGCCCG		
*CXCR*1	F: GCTGACCTGCTCTTCGCCATG	160	60
	R: GGTAGCGGTCCATGCTGATGC		
*POLR*2I	F: AGTGGCAGGACTGGAGTTCGG	145	60
	R: GGTCTTCGGTTCGCGGCAAC		
*RPS*20	F: CCAGCCGCAACGTGAAGTCTC	81	60
	R: GCCGACCTCGCTCAGAACAAG		
oar-miR-433-3p	F: AACGGCATCATGATGGGCTCC	65	60
	R: ATCCAGTGCAGGGTCCGAGG		
oar-miR-200c	F: AAGCGCCTTAATACTGCCGGG	65	60
	R: ATCCAGTGCAGGGTCCGAGG		
oar-mir-221-p5	F: CCTGCTGGACCTGGCATACAAT	65	60
	R: ATCCAGTGCAGGGTCCGAGG		
*U*6	F: ATCCAGTGCAGGGTCCGAGG	65	60
	R: TGAAGCGTGCTCGCTTCGGC		

Table S2 other ceRNA networks information

RNA_class	RNA	miRNA	mRNA
lncRNA	MSTRG.25146	bta-miR-146a	ST3GAL3
lncRNA	MSTRG.23944	bta-miR-146a	ST3GAL3
lncRNA	MSTRG.25507	bta-miR-146a	ST3GAL3
lncRNA	XR_006056307	bta-miR-146a	ST3GAL3
lncRNA	XR_009599878	bta-miR-146a	ST3GAL3
lncRNA	XR_009601615	bta-miR-146a	ST3GAL3
lncRNA	MSTRG.4522	bta-miR-146a	ST3GAL3
lncRNA	MSTRG.1457	bta-miR-146a	ST3GAL3
lncRNA	MSTRG.19452	bta-miR-146a	ST3GAL3

lncRNA	MSTRG.20559	bta-miR-146a	ST3GAL3
lncRNA	MSTRG.10033	bta-miR-146a	ST3GAL3
circRNA	ciRNA345	bta-miR-146a	ST3GAL3
circRNA	ciRNA531	bta-miR-146a	ST3GAL3
circRNA	circRNA24031	bta-miR-146a	ST3GAL3
circRNA	circRNA3361	bta-miR-146a	ST3GAL3
circRNA	circRNA4187	bta-miR-146a	ST3GAL3
lncRNA	MSTRG.26938	chi-miR-128-5p	CSNK1E
lncRNA	MSTRG.5054	chi-miR-128-5p	CSNK1E
lncRNA	MSTRG.23438	chi-miR-128-5p	CSNK1E
lncRNA	MSTRG.16719	chi-miR-128-5p	CSNK1E
lncRNA	MSTRG.27726	chi-miR-128-5p	CSNK1E
lncRNA	XR_009597341	chi-miR-128-5p	CSNK1E
lncRNA	MSTRG.25507	chi-miR-128-5p	CSNK1E
lncRNA	XR_009598835	chi-miR-128-5p	CSNK1E
lncRNA	XR_001039063	chi-miR-128-5p	CSNK1E
lncRNA	MSTRG.10061	chi-miR-128-5p	CSNK1E
lncRNA	XR_003589989	chi-miR-128-5p	CSNK1E
lncRNA	XR_003590528	chi-miR-128-5p	CSNK1E
lncRNA	XR_009595175	chi-miR-128-5p	CSNK1E
lncRNA	LOC121816292:XR_006055805.2	chi-miR-128-5p	CSNK1E
lncRNA	LOC121816326:XR_009595913.1	chi-miR-128-5p	CSNK1E
lncRNA	LOC121816813:XR_009596788.1	chi-miR-128-5p	CSNK1E
lncRNA	LOC121817654:XR_009598106.1	chi-miR-128-5p	CSNK1E
lncRNA	LOC121818498:MSTRG.28814.13	chi-miR-128-5p	CSNK1E
lncRNA	XR_009596487	chi-miR-128-5p	CSNK1E
lncRNA	XR_009596235	chi-miR-128-5p	CSNK1E
lncRNA	XR_009596480	chi-miR-128-5p	CSNK1E
lncRNA	MSTRG.4632	chi-miR-128-5p	CSNK1E
lncRNA	MSTRG.22766	chi-miR-128-5p	CSNK1E
lncRNA	PBX1:MSTRG.1457.6	chi-miR-128-5p	CSNK1E
lncRNA	MSTRG.10127	chi-miR-128-5p	CSNK1E
lncRNA	MSTRG.25055	chi-miR-128-5p	CSNK1E
lncRNA	MSTRG.20782	chi-miR-128-5p	CSNK1E
circRNA	circRNA17219	chi-miR-128-5p	CSNK1E
circRNA	circRNA23125	chi-miR-128-5p	CSNK1E
circRNA	circRNA6141	chi-miR-128-5p	CSNK1E

lncRNA	MSTRG.16719	eca-mir-1543-p5	MOB1B
lncRNA	XR_009599828	eca-mir-1543-p5	MOB1B
lncRNA	MSTRG.22362	eca-mir-1543-p5	MOB1B
lncRNA	XR_009601392	eca-mir-1543-p5	MOB1B
lncRNA	XR_009600443	eca-mir-1543-p5	MOB1B
lncRNA	MSTRG.5600	eca-mir-1543-p5	MOB1B
lncRNA	XR_006059126	eca-mir-1543-p5	MOB1B
lncRNA	XR_006060494	eca-mir-1543-p5	MOB1B
lncRNA	XR_009595885	eca-mir-1543-p5	MOB1B
lncRNA	MSTRG.22834	eca-mir-1543-p5	MOB1B
lncRNA	XR_006060450	eca-mir-1543-p5	MOB1B
lncRNA	XR_009601441	eca-mir-1543-p5	MOB1B
lncRNA	XR_009597424	eca-mir-1543-p5	MOB1B
lncRNA	MSTRG.25525	eca-mir-1543-p5	MOB1B
lncRNA	XR_009599331	eca-mir-1543-p5	MOB1B
lncRNA	MSTRG.1457	eca-mir-1543-p5	MOB1B
lncRNA	MSTRG.5516	eca-mir-1543-p5	MOB1B
lncRNA	MSTRG.10132	eca-mir-1543-p5	MOB1B
circRNA	circRNA116	eca-mir-1543-p5	MOB1B
circRNA	circRNA13608	eca-mir-1543-p5	MOB1B
circRNA	circRNA24861	eca-mir-1543-p5	MOB1B
lncRNA	MSTRG.20208	PC-5p-38445_94	PAK1
lncRNA	MSTRG.27922	PC-5p-38445_94	PAK1
lncRNA	MSTRG.25146	PC-5p-38445_94	PAK1
lncRNA	MSTRG.19683	PC-5p-38445_94	PAK1
lncRNA	MSTRG.28021	PC-5p-38445_94	PAK1
lncRNA	MSTRG.17399	PC-5p-38445_94	PAK1
lncRNA	MSTRG.5889	PC-5p-38445_94	PAK1
lncRNA	MSTRG.17589	PC-5p-38445_94	PAK1
lncRNA	MSTRG.5625	PC-5p-38445_94	PAK1
lncRNA	MSTRG.20482	PC-5p-38445_94	PAK1
lncRNA	MSTRG.20765	PC-5p-38445_94	PAK1
lncRNA	MSTRG.9619	PC-5p-38445_94	PAK1
lncRNA	MSTRG.22782	PC-5p-38445_94	PAK1
lncRNA	MSTRG.20628	PC-5p-38445_94	PAK1
lncRNA	MSTRG.21765	PC-5p-38445_94	PAK1
lncRNA	MSTRG.25578	PC-5p-38445_94	PAK1
lncRNA	MSTRG.27996	PC-5p-38445_94	PAK1
lncRNA	XR_009597400	PC-5p-38445_94	PAK1
lncRNA	MSTRG.23556	PC-5p-38445_94	PAK1
lncRNA	MSTRG.5551	PC-5p-38445_94	PAK1

lncRNA	MSTRG.22766	PC-5p-38445_94	PAK1
lncRNA	MSTRG.9561	PC-5p-38445_94	PAK1
lncRNA	MSTRG.1457	PC-5p-38445_94	PAK1
lncRNA	MSTRG.19452	PC-5p-38445_94	PAK1
lncRNA	MSTRG.10033	PC-5p-38445_94	PAK1
lncRNA	MSTRG.28365	PC-5p-38445_94	PAK1
lncRNA	MSTRG.5516	PC-5p-38445_94	PAK1
lncRNA	MSTRG.19837	PC-5p-38445_94	PAK1
lncRNA	MSTRG.9702	PC-5p-38445_94	PAK1
lncRNA	MSTRG.19527	PC-5p-38445_94	PAK1
lncRNA	MSTRG.10132	PC-5p-38445_94	PAK1
lncRNA	MSTRG.21804	PC-5p-38445_94	PAK1
lncRNA	MSTRG.22520	PC-5p-38445_94	PAK1
lncRNA	MSTRG.17414	PC-5p-38445_94	PAK1
lncRNA	MSTRG.18125	PC-5p-38445_94	PAK1
circRNA	ciRNA531	PC-5p-38445_94	PAK1
circRNA	ciRNA650	PC-5p-38445_94	PAK1
circRNA	circRNA12870	PC-5p-38445_94	PAK1
circRNA	circRNA16520	PC-5p-38445_94	PAK1
circRNA	circRNA22401	PC-5p-38445_94	PAK1
circRNA	circRNA23501	PC-5p-38445_94	PAK1
circRNA	circRNA24064	PC-5p-38445_94	PAK1
circRNA	circRNA24758	PC-5p-38445_94	PAK1
circRNA	circRNA27270	PC-5p-38445_94	PAK1
circRNA	circRNA3315	PC-5p-38445_94	PAK1
circRNA	circRNA3361	PC-5p-38445_94	PAK1
circRNA	circRNA3639	PC-5p-38445_94	PAK1
circRNA	circRNA4187	PC-5p-38445_94	PAK1
lncRNA	MSTRG.16276	hsa-miR-320c	RASSF2
lncRNA	MSTRG.17937	hsa-miR-320c	RASSF2
lncRNA	MSTRG.28021	hsa-miR-320c	RASSF2
lncRNA	MSTRG.5056	hsa-miR-320c	RASSF2
lncRNA	MSTRG.5889	hsa-miR-320c	RASSF2
lncRNA	MSTRG.17589	hsa-miR-320c	RASSF2
lncRNA	MSTRG.20765	hsa-miR-320c	RASSF2
lncRNA	MSTRG.20628	hsa-miR-320c	RASSF2
lncRNA	MSTRG.25119	hsa-miR-320c	RASSF2
lncRNA	MSTRG.21767	hsa-miR-320c	RASSF2
lncRNA	XR_009598755	hsa-miR-320c	RASSF2
lncRNA	XR_003586620	hsa-miR-320c	RASSF2
lncRNA	MSTRG.10061	hsa-miR-320c	RASSF2

lncRNA	XR_009595272	hsa-miR-320c	RASSF2
lncRNA	XR_003590528	hsa-miR-320c	RASSF2
lncRNA	XR_009595175	hsa-miR-320c	RASSF2
lncRNA	XR_006055599	hsa-miR-320c	RASSF2
lncRNA	XR_009595913	hsa-miR-320c	RASSF2
lncRNA	XR_009596553	hsa-miR-320c	RASSF2
lncRNA	XR_009600509	hsa-miR-320c	RASSF2
lncRNA	XR_009595103	hsa-miR-320c	RASSF2
lncRNA	XR_009595800	hsa-miR-320c	RASSF2
lncRNA	XR_009596732	hsa-miR-320c	RASSF2
lncRNA	XR_009596972	hsa-miR-320c	RASSF2
lncRNA	XR_009596974	hsa-miR-320c	RASSF2
lncRNA	MSTRG.22467	hsa-miR-320c	RASSF2
lncRNA	XR_009597423	hsa-miR-320c	RASSF2
lncRNA	XR_009597751	hsa-miR-320c	RASSF2
lncRNA	MSTRG.25301	hsa-miR-320c	RASSF2
lncRNA	XR_009600754	hsa-miR-320c	RASSF2
lncRNA	XR_009600755	hsa-miR-320c	RASSF2
lncRNA	MSTRG.5551	hsa-miR-320c	RASSF2
lncRNA	MSTRG.14855	hsa-miR-320c	RASSF2
lncRNA	MSTRG.17253	hsa-miR-320c	RASSF2
lncRNA	MSTRG.1457	hsa-miR-320c	RASSF2
lncRNA	MSTRG.19452	hsa-miR-320c	RASSF2
lncRNA	MSTRG.23327	hsa-miR-320c	RASSF2
lncRNA	MSTRG.17610	hsa-miR-320c	RASSF2
lncRNA	MSTRG.609	hsa-miR-320c	RASSF2
lncRNA	MSTRG.20494	hsa-miR-320c	RASSF2
lncRNA	MSTRG.22520	hsa-miR-320c	RASSF2
lncRNA	MSTRG.25669	hsa-miR-320c	RASSF2
lncRNA	MSTRG.21190	hsa-miR-320c	RASSF2
lncRNA	MSTRG.28016	hsa-miR-320c	RASSF2
circRNA	ciRNA345	hsa-miR-320c	RASSF2
circRNA	ciRNA650	hsa-miR-320c	RASSF2
circRNA	circRNA13608	hsa-miR-320c	RASSF2
circRNA	circRNA29802	hsa-miR-320c	RASSF2
circRNA	circRNA3361	hsa-miR-320c	RASSF2
circRNA	circRNA9772	hsa-miR-320c	RASSF2
